# In vivo identification of the retinal layer containing photopigments in OCT images through correlation with two-photon psychophysics

**DOI:** 10.1038/s41598-024-65234-7

**Published:** 2024-07-04

**Authors:** Maciej M. Bartuzel, Alejandra Consejo, Patrycjusz Stremplewski, Marcin Sylwestrzak, Maciej Szkulmowski, Iwona Gorczynska

**Affiliations:** 1 Institute of Physics, Faculty of Physics, Astronomy and Informatics, Nicolaus Copernicus University in Torun, Toruń, Poland; 2 Aragon Institute for Engineering Research (I3A), University of Zaragoza, Zaragoza, Spain

**Keywords:** Retina, Imaging and sensing, Biomedical engineering

## Abstract

Two-photon vision enables near-infrared light perception in humans. We investigate the possibility to utilize this phenomenon as an indicator of the location of the outer segments of photoreceptor cells in the OCT images. Since two-photon vision is independent on OCT imaging, it could provide external to OCT reference relative to which positions of retinal layers visible in OCT imaging could be measured. We show coincidence between OCT imaging of outer retinal layers and two-photon light perception. The experiment utilizes an intrinsic nonlinear process in the retina, two-photon absorption of light by visual photopigments, which triggers perception of near-infrared light. By shifting the focus of the imaging/stimulus beam, we link the peak efficiency of two-photon vision with the visibility of outer segments of photoreceptor cells, which can be seen as in vivo identification of a retinal layer containing visual photopigments in OCT images. Determination of the in-focus retinal layer is achieved by analysis of *en face* OCT image contrast. We discuss experimental methods and experimental factors that may influence two-photon light perception and the accuracy of the results. The limits of resolution are discussed in analysis of the one-photon and two-photon point spread functions.

## Introduction

The invention of optical coherence tomography (OCT) revolutionized ophthalmic care by permitting non-invasive, three-dimensional imaging of the laminar structure of the living human retina^1^. Since OCT images do not reveal details of retinal structures at the subcellular level like in vitro microscopic images do, the anatomic attributions of distinct hypo- and hyper-reflective bands visible in typical OCT B-scans were originally assigned by correlation with histological studies in animals^2,3^. To our knowledge, the only comparative study using human eyes in vitro was reported in 2006 by Chen et al^4^. Advancements in OCT technology in multiple research centers around the world elevated the quality of the images and led to multiple improvements, including ability to resolve more layers^5–7^. Investigators labeled them providing hypothetical attributions to retinal structures, but due to technical differences among the experimental systems and signal processing algorithms, inconsistencies arose among these attributions and associated nomenclature.

As OCT systems were rapidly commercialized and adopted in clinical settings, the diversity in nomenclature usage among investigators became more pronounced, underscoring the need for a standardized approach to describe OCT B-scan structures. In response, a panel of retina specialists was convened in 2014 to review and standardize OCT retinal imaging terminology^8^. Although some OCT bands/histology correlations have achieved widespread acceptance within the community, others continue to provoke lively discussions and controversy^9–13^.

Correlative histological studies remain the primary method for linking OCT bands to the retinal microstructures they represent; however, this technique faces inherent difficulties. Precise alignment between the micrograph and OCT B-scan is critical. Since OCT measures optical path, the results may be affected by the variation of reported refractive indices of retinal layers^10,14^. Histological sample preparation might also result in artifacts, for instance, due to tissue deformations^10^.

In instances of uncertainty, the integration of complementary methods offers a path to corroborative validation. For example, polarization-sensitive OCT (PS-OCT) may provide additional contrast for discerning the retinal nerve fiber layer (RNFL) due to its birefringence^15^ or retinal pigment epithelium (RPE) due to its depolarization properties related to the presence of melanin pigments^16^. Outer nuclear layer (ONL) and Henle fiber layer (HFL) may be accurately differentiated with a purposeful alteration of the OCT beam entry position, sometimes referred to as directional OCT (D-OCT)^17^. Techniques that simultaneously probe both structure and function of the retina may also be useful. A prominent example of such a method is OCT optoretinography (ORG), which has been extensively investigated in recent years^18–23^. These functional studies hold promise for in vivo probing of the photoreceptor layer, in which particularly contentious outer retinal OCT bands 2 and 3 are located^11,14,24,25^. Outer retinal bands 2 and 3 are frequently associated with Inner Segment / Outer Segment (IS/OS) junction of photoreceptor cells and Cones Outer Segment Tips (COST) respectively^11,24^. Here, for consistency, we also adopted this nomenclature.

**Figure 1:**
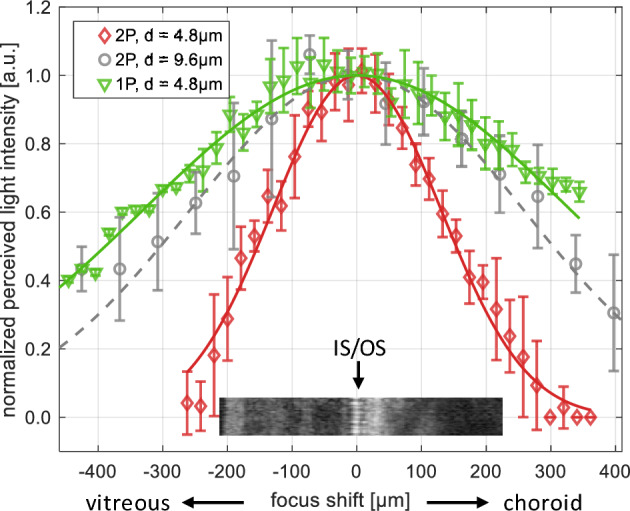
Comparison of the decrease of light intensity perceived in two-photon (2P) and one-photon (1P) vision with defocus, dependent on the wavelength of stimulus light and the focusing of the light beam on the retina, as observed in Subject 2. OCT B-scan inset serves as a guide for approximate focus location in the retina. The red plot represents 2P vision with 1060 nm stimulus light, a 4.8 µm nominal beam spot diameter at the retina, and a 153 µm half-width at half maximum (HWHM) of the Gaussian function fit ($\text {r}^2=0.98$). The gray plot depicts 2P vision with 1060 nm stimulus light, a 9.6 µm nominal beam spot diameter at the retina, and a 304 µm HWHM of the Gaussian function fit ($\text {r}^2=0.73$). The green plot illustrates one-photon (1P) vision with a 520 nm stimulus light, a 4.8 µm nominal beam spot diameter at the retina, and a 389 µm HWHM of the Gaussian function fit ($\text {r}^2=0.96$).

The outer segments of photoreceptor cells, which lay between IS/OS junction and COST, contain visual photopigments. This is the location of the retina where the visual process is initiated. Under normal conditions, the energy from one photon (1P) of visible light can trigger the isomerization of the chromophore (11-*cis*-retinylidene), a key component of photopigment protein. This conformational change in the molecule allows for the activation of the G protein, which in turn mediates an enzymatic signaling cascade leading to an electrical response in the photoreceptor cell^26^. However, under specific conditions, humans can also experience infrared vision. Reports regarding this effect date back to the 1950s^27–29^, but the mechanism behind this phenomenon was only recently explained through two-photon (2P) chromophore isomerization^30^, giving rise to the term “2P vision”^31–34^. While the efficiency of 1P absorption is linearly dependent on light intensity, the efficiency of 2P absorption depends on the square of light intensity^35^. Thus, when the focus of the light beam is shifted away from the layer containing photopigments, the perceived light intensity in 2P vision decreases much more steeply than in 1P vision^33,36–39^.

The 2P light perception intensity can be assessed in vivo through psychophysics method of adjustment experiments. When the focus of a near-infrared (NIR) light beam entering the eye is altered, the perceived light intensity changes correspondingly, reaching its maximum when the 2P vision is most efficient, that is, when the beam is focused on the layer that maximizes chromophore isomerization. In OCT, the quality of the three-dimensional images changes axially (in depth) with applied focal shifts. A metric can be established to quantify the quality of OCT *en face* projections as the focus is shifted through the depth of the retina. The peak of this metric value pinpoints the in-focus position of a specific OCT *en face* image. As the same light beam is used for OCT imaging and for the psychophysics experiment we can then compare the results from both experiments.

In this study, we investigate the possibility to utilize the 2P absorption in retinal photopigments as an indicator of the location of the outer segments of photoreceptor cells in the OCT images. Since 2P absorption phenomenon is independent on OCT imaging, it could provide additional and external to OCT reference relative to which positions of retinal layers visible in OCT imaging could be measured, potentially reducing ambiguities in their identification. While exploring this concept we have also encountered and identified several factors which can adversely affect experiments involving 2P vision as a study subject or as a tool to research other phenomena. We show correlation between the location of OCT beam focus on specific retinal layers and the 2P light perception intensity in the living human eye. We demonstrate an experimental method to determine the spatial location of photopigments in three-dimensional OCT retinal images. We discuss experimental factors influencing 2P light perception which can serve as experiment-based guidelines for studies involving this phenomenon. And we analyze theoretical resolution limits in 2P psychophysics experiments and in the determination of focus position in OCT imaging.

## Results

**Figure 2:**
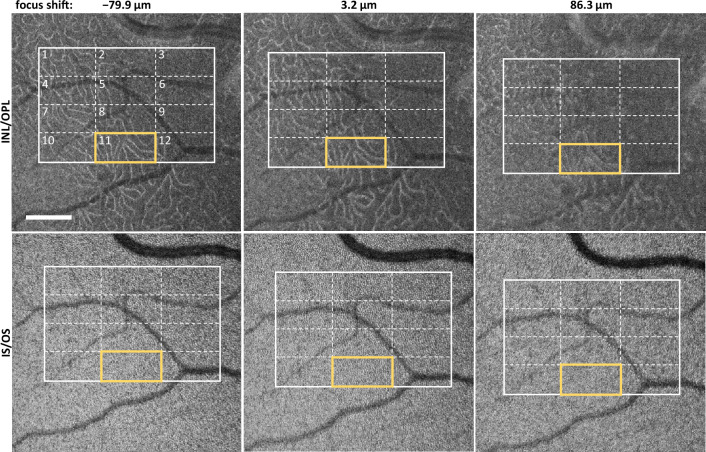
*En face* projections from the perifovea of Subject 1, obtained at different focal shifts. Analyzed region of interest (ROI) is delineated by a thick white frame and is subdivided into twelve subregions. The ROI_11_, outlined in yellow, is shown in more detail in Fig. 3 at the depths of the INL/OPL, IS/OS junctions, and COST layers. The given focal shift values are relative to the maximum position on the normalized IS/OS contrast curve, as determined analytically (see Fig. 6). The scale bar indicates $1^\circ$ field of view.

In the psychophysics experiments, human subjects were exposed to a 2P light stimulus formed by a scanning beam of NIR light (1060 nm) perceived by all subjects as green horizontal line. When we introduced focal shift by decollimating the beam entering the eye, subjects reported a change in the perceived light intensity. As demonstrated in Fig. 1 (red circles), 2P sensitivity is highest at a certain optimum focal plane and decreases as focus is shifted in either direction. In this subject, a focal shift of $\sim 150$ µm (0.5 diopters) decreased the perceived light intensity by half. When the shift exceeded $\sim 200$ µm (0.7 diopters), the light became imperceptible to the subject. For reference, the mean thickness of the retina is approximately 200–270 µm^40^. In a similar experiment, when the NIR light was replaced with green light (520 nm), the decrease in perceived light intensity due to beam defocus was slower, and the perception never ceased (Fig. 1, green diamonds). Instead, as expected, the subjects reported a substantial broadening of the light line due to defocus (“blurring”). In contrast to visible light, the perception of the NIR 2P stimulus faded so quickly with increasing defocus that the subjects were barely able to notice any blurring before the light became imperceptible. In this study, our interest lies not in the specific sensitivity values of the human visual system. Instead, we are interested in how well-defined the peaks are, i.e., how narrow the distributions are, as this determines the resolution of our results. Consequently, in Fig. 1 we normalized the plots to their respective maxima and shifted them so that their maxima overlapped.

Figure 1 also illustrates the narrowing of the 2P perceived light intensity graphs when the light beam is focused into a smaller spot on the retina. The distribution marked in gray was obtained using L’_3_ imaging lens (Methods) with a focal length of ${\text {f}_{\text {L3'}} = 75\,~\text {mm}}$. In this setup, the nominal diffraction-limited spot diameter at the retina is ${\text {d}_{1/\text {e}^2}^{(\text {2P,f75})}=9.6}$ µm. This corresponds to ${\Delta \text {z}_{\text {HWHM}}^{(\text {2P,f75})}=304}$ µm half-width at half-maximum (HWHM) of the fitted Gaussian function shown in Fig. 1 as dashed line. The distribution marked in red was obtained using an L_3_ imaging lens with a focal length of ${\text {f}_{\text {L3}}=150\, \text {mm}}$, yielding a nominal beam spot diameter at the retina of ${\text {d}_{1/\text {e}^2}^{(\text {2P,f150})}=4.8\,\upmu \text {m}}$. This corresponds to ${\Delta \text {z}_{\text {HWHM}}^{(\text {2P,f150})}=153}$ µm HWHM of the fitted Gaussian function (red line). These values serve here as an indication of the focusing performance of the two imaging setups used in our experiments.

**Figure 3:**
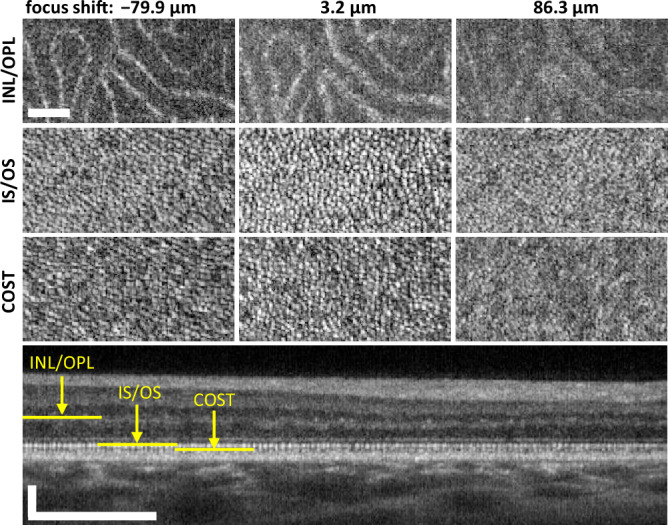
ROI_11_ (Fig. 2) at the depth of INL/OPL, IS/OS junctions and COST layer as indicated in the B-scan (bottom row). The INL/OPL junction appears blurred in the middle column, whereas the IS/OS junction and COST layer are in focus, as evident from the visibility of the cone photoreceptors mosaic. Focus had to be shifted to sharpen the images of the deep capillary plexus located in the INL/OPL junction (left column). The applied focal shift approximately matches the physical distance between the INL/OPL and IS/OS junctions. Scale bars: ROI—$0.25^\circ$; B-scan, transverse—$1^\circ$; Bscan, axial—100 µm.

**Figure 4:**
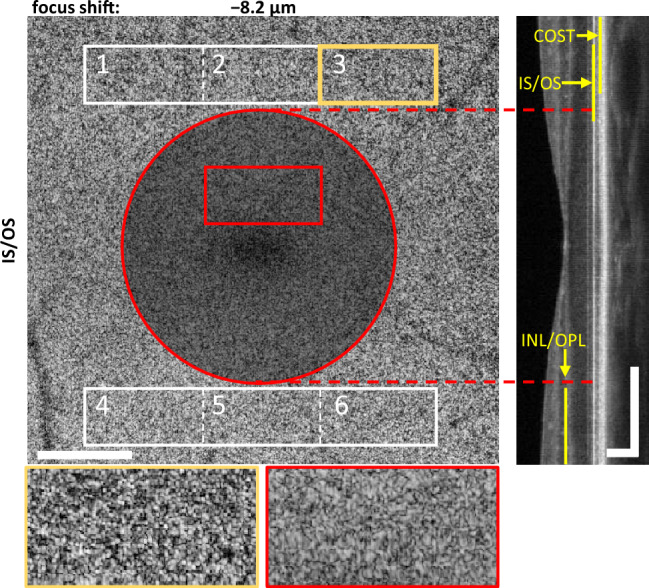
OCT *en face* image of the IS/OS junction in the fovea and parafovea indicating regions of interest included in image contrast calculations (ROIs 1–6) and excluded from calculations (red outlines). Bottom insets—zoomed-in regions of interest from the inclusion area (yellow outline—photoreceptors mosaic is discernible) and from the excluded area (red outline – only speckle noise is visualized). Right inset—B-scan indicating depths at which *en face* images were generated. Scale bars: *en face*—$1^\circ$; Bscan, transverse—$1^\circ$; Bscan, axial—100 µm.

Figure 2 presents OCT *en face* projections acquired in the perifovea at three different focal shifts. The top row shows the junction between INL and OPL layers (INL/OPL), visualizing the vasculature of the deep capillary plexus. The bottom row presents the IS/OS junction, corresponding to the *en face* projection at the depth of the 2nd outer retinal band. Given the depth of focus (DoF = 34 µm) of our system (Methods), these different retinal layers come into focus with distinct focal shift settings. As an example, the region of interest 11 (ROI_11_), indicated by a yellow rectangle in Fig. 2 and displayed in Fig. 3, reveals sharp images of the outer capillary plexus vessels and the photoreceptor cells mosaics when these layers are in their respective focus. The focus of the INL/OPL is about $-77$ µm ($-0.3$ dioptres) from the IS/OS focus. The sharpest images of the IS/OS and COST photoreceptor mosaics are obtained when they are at similar focus positions. As the amount of defocus increases, the vessels start to appear blurred, and distortions in the photoreceptor mosaics become evident.

Figure 4 presents an OCT *en face* projection of the fovea and parafovea with the focus set at the IS/OS junction. Due to the unique anatomy of the fovea we chose the regions of interest differently from those in the perifovea. The area around the foveal pit (red circle in Fig. 4) was excluded from the analysis due to lack of visibility of distinct features that could contribute to the image quality metric. The two insets demonstrate that no features other than speckles are visualized within the excluded region (Fig. 4, red rectangle). In contrast, features resembling the photoreceptors mosaic become evident in areas included in the analysis (parafovea), such as ROI_3_ (Fig. 4, yellow rectangle).

**Figure 5:**
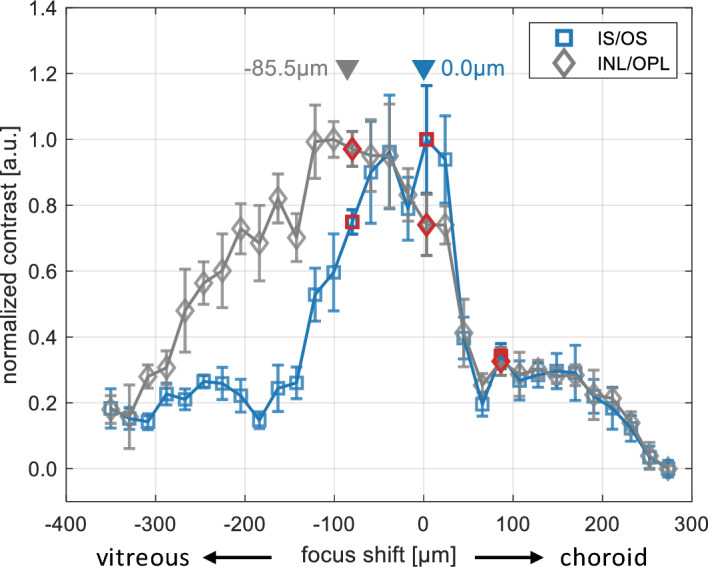
OCT contrast graphs of the IS/OS and INL/OPL junctions. The origin of the abscissa is set to align with the focal position yielding maximum contrast in the IS/OS junction *en face* images. Negative values denote a focal shift towards the inner retina, positive values indicate a shift towards RPE. Focal shifts corresponding to the images shown in Figs. 2 and 3 are denoted by red markers.

Preliminary estimations of optimal focal shifts can be obtained through visual judgment of captured images. Nonetheless, these estimations should be congruent with values derived from the image quality metric, which provides a more precise indication of optimal focus. For twelve ROIs indicated in Fig. 2 and six ROIs from Fig. 4, we calculated image contrasts and plotted them against focal shift. Figure 5 shows an example of the contrast graphs for the IS/OS (blue) and INL/OPL (gray) junctions, calculated for ROI_11_. Based on the graphs, an estimated focal shift of $-85.5$ µm (or $-0.3$ dioptres) is needed to bring the INL/OPL junction into focus. The depth-separation between the INL/OPL and IS/OS OCT *en face* projections is $\Delta {}\text {z}=93.6$ µm. The difference of 8.7 µm between these values is below the depth of focus (DoF) of our system. This close alignment of the focal shift between the two layers, as determined from image contrast graphs and their physical separation directly measured from OCT images, provides a reasonable assurance of our methodology’s credibility.

The perifoveal OCT *en face* projections of the IS/OS junction and COST layer render visibility of the photoreceptor mosaics in all 12 ROIs. A mosaic-like appearance can also be detected in ROIs 1–6 selected in the parafoveal region, outside of the foveal pit as presented in Fig. 4. Additionally, the contrast graphs maintain similar maxima positions across all ROIs in both perifoveal and parafoveal areas. To facilitate the next stage of the analysis, we performed contrast calculations within the entire area indicated by a solid, bold outline in Fig. 2, and the region covered by ROIs 1–6 in Fig. 4. The objective of this analysis is to ascertain if the peaks of the contrast graphs derived from OCT imaging correspond with the 2P light intensity perception maxima obtained from 2P psychophysics experiments.

**Figure 6:**
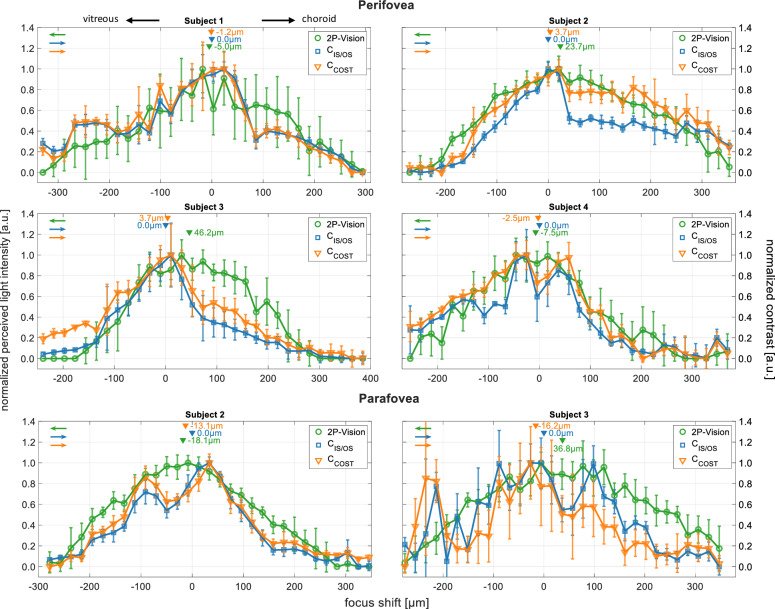
Comparative analysis of two-photon light perception intensity (green) and OCT imaging contrast of IS/OS junction (blue) and COST layer (orange) over 31 focal positions in the perifoveal and parafoveal regions of the retina. The origin of the abscissa is defined by the focus position yielding the maximum contrast in IS/OS junction images. Negative focal shifts are oriented towards the inner retina. Positive values extend towards the retinal pigment epithelium (RPE). Data points represent the mean values at each focus position, with error bars indicating 95% confidence intervals. Downward pointing arrowheads denote peak positions obtained from parabolic function fits (Methods). Arrows in matching colors indicate the ordinates corresponding to each data series.

Figure 6 presents data from the perifoveal region of four subjects and from the parafoveal region in two subjects. The 2P perceived normalized light intensity graphs are depicted in green, with peak localizations highlighted by downward-facing green arrowheads. Peak positions, determined by the maxima of fitted parabolic functions (Methods), are summarized in Table 1. 2P perceived light intensity diminishes to half the maximum value with a focal shift of $\sim 78\upmu \text {m}$ to $142\,\upmu \text {m}$ from the peak position, subject to individual variations (Table 2). These values are derived as the half-width at half-maximum (HWHM) of Gaussian functions fitted to the data points. Figure 1 illustrates the Gaussian function fit applied to data from Subject 2. For Subjects 2–4, the coefficients of determination (r^2^) range between 0.92 and 0.99, while for Subject 1, the value is 0.89.

**Table 1:** Locations of the maxima in 2P light perception graphs and OCT imaging contrast graphs of COST layer. Reported values are relative to the focus position resulting in the maximum contrast of the IS/OS junction.

	2P vision peak (µm)	COST (µm)	$\Delta$ (µm)
Perifovea			
Subject 1	− 5.0	− 1.2	5.0
Subject 2	23.7	3.7	23.7
Subject 3	46.2	3.7	46.2
Subject 4	− 7.5	− 2.5	7.5
Parafovea			
Subject 1	− 18.1	− 13.1	− 18.1
Subject 2	36.8	− 16.2	53

$\Delta$ – distance between two most separated peak locations.

Figure 6 also showcases normalized image contrast graphs for the IS/OS junction (blue) and COST layer (orange), obtained from the perifoveal and parafoveal regions of the retina. The peak positions are indicated by downward-facing arrowheads in colors corresponding to the OCT imaging contrast graphs. Upon visual inspection, the graphs corresponding to the IS/OS junction and COST layer exhibit similar shapes within each subject, and their peak positions also appear to align. In our analysis, we determine the positions of the maxima for all graphs in Figure 6 through data smoothing and localizing the parabolic fit maximum, as outlined in Methods. The results are summarized in Table 1. The peak positions are given relative to the focus positions at which the maximum contrast of the IS/OS junction was determined in each subject. To clarify, the values given for the COST layer represent the distances between focus positions that yield the maximum image contrasts for the COST and IS/OS layers. In several instances, the identified peak position for the COST layer precedes the IS/OS junction image contrast peak, which contradicts the known anatomical arrangement of these layers. However, the largest distance between these peak positions is $16.2\,\upmu \text {m}$, which is below the system’s DoF. Thus, we conclude that the contrast peak positions of the IS/OS junction and the COST layer are effectively indistinguishable in this analysis. The data in Table 1 indicates that the differences in peak positions between the contrast graphs for the IS/OS junction, COST layer, and the 2P light perception graphs fall within the DoF of our experimental setup. However, an exception is noted for Subject 3, who presents a slight degree of astigmatism (0.25 dioptres) and mild myopia ($-1.5$ dioptres). In this specific case, the separation between the peak of the 2P perception graph and the peak of the IS/OS junction image contrast exceeds the system’s DoF by $\sim 12\,\upmu \text {m}$ in the perifovea and $\sim 3\,\upmu \text {m}$ in the parafovea. Nevertheless, our results confirm the alignment of two-photon vision with the location of the outer segments of the photoreceptor cells. This alignment is within the resolutions of our experimental setup in the 2P psychophysics experiment and in OCT imaging.

## Discussion

Our experiments demonstrate a collocation of peak light perception in 2P vision and outer retinal bands 2 and 3 visualized in OCT imaging. Band 2 is typically identified as the junction between the inner and outer segments of photoreceptor cells (IS/OS). Band 3 is linked to the cone outer segment tips (COST). Between these bands photoreceptor outer segments are located in OCT images. Our results show that the visual system reacts to the stimulus (subjects report seeing light) when the beam of NIR light is focused on the outer segments of photoreceptor cells. The experiment links the peak efficiency of 2P vision with the outer segments of photoreceptor cells. The findings represent an experimental, in vivo identification of a retinal layer containing visual photopigments in OCT images.

The experiment hinges on an intrinsic nonlinear process that takes place in the retina, 2P absorption of light by visual photopigments. This process involves a simultaneous absorption of two photons of light, not necessarily of equal energies. However, the sum of their energies must match the absorption energy of the photoreceptor proteins’ chromophore (11-*cis*-retinylidene), triggering its isomerization and thereby initiating the cascade of processes that result in light perception^30^. This process should not be confused with 2P fluorescence, where the absorption of two photons of light leads to emission of a single photon of higher energy. In 2P vision two photons are absorbed but no light is emitted.

2P absorption isomerization rate, or the number of isomerized molecules per second, is proportional to the square of the light intensity ($I^2$), reflecting the inherently lower probability of simultaneous photon interactions, compared to 1P absorption, which is proportional to *I*^41^. As the absorption spectrum of chromophores stretches into the NIR range, the absorption cross-section becomes negligible beyond 900 nm. Consequently, 2P absorption rates might exceed those of 1P if photon flux is sufficiently increased. This is particularly pronounced in the spectral region where the 1P absorption cross-section is low, yet the wavelength reaches twice the value of the chromophore’s absorption peak. Notably, at around 900 nm, as the wavelength of a laser source increases, a perceptual shift from red to purple, then blue, can be observed. This marks the transition from a region dominated by 1P absorption to one where 2P absorption prevails^42^.

Visual thresholds, quantifying the minimum light amount necessary to trigger perception, can be used to gauge the human eye’s sensitivity to specific light wavelengths. Recent studies have reported that the power of light entering the eye required to trigger perception of ${1045\,\text {nm}}$ wavelength light (2P vision) was about ${25\,\upmu \text {W}}$, while the threshold for ${522.5\,\text {nm}}$ wavelength light (1P vision) was approximately ${25\,\text {fW}}$ ($10^9$ times less)^43,44^. These values should be interpreted as approximations, as 2P absorption efficiency depends on several factors: the focusing of the light beam at the photoreceptor cells’ outer segments, the duration and repetition of the light pulses^45^, and potentially the type of photoreceptors (rods or cones) where absorption takes place.

**Table 2:** Comparison of calculated one-photon (1P), two-photon (2P) transverse and axial intensity point spread functions (PSF) extents and axial spans of the experimentally obtained two-photon vision intensity distributions.

Calculations results				
	Transverse spot size		Axial span	
1P PSF	r_HWHM_	r$_{1/\text {e}^2}$	$\Delta z$_HWHM_	$\Delta z_{1/\text {e}^2}$
Gaussian beam	1.41 µm	2.4 µm	17.1 µm	43.2 µm
Airy distribution	1.35 µm	2.13 µm	22.7 µm	35.7 µm
2P PSF				
Gaussian beam	1 µm	1.7 µm	11 µm	22.4 µm
Airy distribution	0.94 µm	1.58 µm	16.3 µm	26.7 µm
Axial 2P PSF convolved with “top-hat” function				
Gaussian beam	–	–	17.5 µm	29.9 µm
Airy distribution	–	–	19.2 µm	31.6 µm

Experimental results				
Subjects	S1	S2	S3	S4
	Axial span,$\Delta z$_HWHM_			
Perifovea	176 µm	185 µm	123 µm	134 µm
Parafovea	–	153 µm	220 µm	-

Beam waist radius of the collimated Gaussian beam at the cornea: 2.4 mm. Eye pupil radius used for Airy distribution calculations: 3.5 mm. Width of the “top-hat” function: 30 µm.$\text {r}$—beam spot radius in the focal plane,$\Delta z$—axial focus span along the optical axis, HWHM - half width at half maximum, and$1/\text {e}^2 \approx 0.135$of peak intensity.

In our psychophysics experiment, we take advantage of the spatial confinement of 2P absorption to areas of high photon flux, achievable by focusing the beam of NIR light at the location of interest in the retina. Fig. 1 illustrates how quickly the light perception deteriorates as the light beam defocuses from the outer segments of photoreceptor cells. This deterioration is dependent on the wavelength of light and the spot size of the focused light beam. The advantage of using 2P vision in our experiment is clear. The light perception intensity in 2P vision as a function of defocus has narrower distribution (HWHM of the fitted Gaussian function ${\Delta \text {z}_{\text {HWHM}}^{(\text {2P,f150})}=153}$ µm) as compared to 1P vision intensity plot (${\Delta \text {z}_{\text {HWHM}}^{(\text {1P,f150})}=389}$ µm). Our results further illustrate the benefits of utilizing higher resolution imaging setups, as represented by the plot marked in red versus the one in gray (Fig. 1). The intensity distribution is narrower in the higher resolution setup (${\text {f}_{\text {L3}}=150\, \text {mm}}$) compared to the lower resolution setup (${\text {f}_{\text {L3'}}=75\, \text {mm}}$), with HWHM values of ${\Delta \text {z}_{\text {HWHM}}^{(\text {2P,f75})}=304}$ µm. This enhancement in spatial (specifically axial, i.e., along the beam of light) localization of 2P vision is anticipated, given that 2P absorption depends on photon flux, which in turn depends on the focus of the light beam. The focusing of the light beam can be represented by a three-dimensional point spread function (PSF). This allows us to discuss the resolution limits when determining the location of the retinal layer containing photopigments in a psychophysics experiment where the focus is shifted through the retina’s depth (detailed analysis of PSF is included in Supplementary discussion).

In our experiments, the power of the light entering the eye was ${1.8\, \text {mW}}$ at ${1060\, \text {nm}}$ wavelength, adhering to the maximum permissible exposure defined by safety standards^46^ and is about an order of magnitude higher than the reported 2P visual threshold^44^. The probability that 2P absorption will occur is proportional to the square of the light intensity across the beam focus. In other words, the intensity of the 2P absorption-triggered perception of light is proportional to the square of the 1P PSF^35^, although the 2P PSF might be truncated if the light intensity drops below the visual threshold at a specific defocus.

We should note that our experiment did not incorporate any corrections for aberrations in the imaged eyes. Although cone photoreceptor mosaics were visible in all subjects through OCT imaging of the perifovea using high-resolution imaging setup (${\text {f}_{\text {L3}}=150, \text {mm}}$, beam spot diameter at the cornea ${4.8 \text {mm}}$), indicating good performance of the imaging system and low aberrations in the imaged eyes, aberrations could still potentially influence the results. This influence is one plausible explanation for the varying widths and shapes of the 2P vision graphs seen in Fig. 6 among different subjects and between two retinal locations. Nevertheless, we have observed that more focused beam of light on the photoreceptor outer segments leads to narrower distributions of 2P perceived light intensity^33^. This finding indicates that the use of adaptive optics (AO) could further enhance the results. Indeed, a study of 2P vision that employed a system equipped with an AO correction confirms this hypothesis^38,39^.

Given in Supplementary discussion Equations S8–S11 define the 2P spot sizes – the transverse and axial extents of the volume within which 2P vision can occur if absorbing molecules are present within this volume and the light intensity does not drop below the visual threshold. In our experimental setup, the radii of collimated beam waists entering the eye are 1.2 mm and 2.4 mm (Table 3). The results of calculations of the spot sizes of the beams focused at the retina are given in Table 2. We include calculations for 1P and 2P PSF in case of Gaussian beam imaging and Airy distribution^47^ at the retina. We also take into account the axial distribution of photopigments in the retina. We assume that the photopigments are uniformly distributed within the outer segments of photoreceptor cells ($\sim 30$ µm in length^11^) and the probability of 2P absorption is constant regardless on the location within outer segments. Then this distribution can be represented as a rectangular function and convolved with the PSFs for more accurate calculations of the best-case-scenario resolutions in our experiments. We have numerically computed the convolution of Gaussian and Airy 2P absorption PSFs with a rectangular function. For the Gaussian PSF, we utilized parameters from the high-resolution setup, featuring $\text {DoF}=34$ µm. In the Airy PSF computations we employed eye pupil diameter of ${7\,\text {mm}}$.

The comparison between the axial spans of 2P vision intensity distributions obtained in the psychophysics experiments (${\Delta \text {z}_{\text {HWHM}}}$ in Table 2) and the corresponding axial spans of aberration-free 2P PSFs for Gaussian beam imaging makes it apparent that our experimental setup is not operating at diffraction limit. When comparing the calculated 2P PSFs for the Gaussian beam and the Airy distribution, there is a discernible trade-off between transverse and axial resolutions in the case of diffraction-limited imaging. Implementing a uniform light distribution at the eye pupil (Airy distribution in the beam focus at the retina) slightly enhances transverse resolution over the Gaussian beam illumination. However, if the axial resolution is an important factor in 2P vision experiments, the use of Gaussian beams might prove beneficial, provided the spot diameter of the beam illuminating the cornea is appropriately selected. This benefit arises from the Lorentzian light distribution along the optical axis, which exhibits a narrower span at half maximum of the light intensity peak compared to the Airy distribution.

In our experiment, correction of aberrations would enhance the precision of localizing the light-absorbing layer within the retina. However, aberrations are not the sole source of potential inaccuracies. Eye movements represent another source that can impact experimental results. Large eye motions can induce vignetting of the incoming light beam, leading to increased variability in the subject’s response to the stimulus. This variability arises from alterations in stimulus brightness due to vignetting or, in more severe cases, the total occlusion of light by the iris. Even small eye motions can potentially broaden the axial span of the 2P vision response to focal shifts introduced in the experiment, as they may induce shifts in the position of photoreceptors’ outer segments relative to the focal point. In light of these considerations, it is advisable to at least monitor the position of the pupil during the experiment. To address this, we have employed a pupil-viewing camera in our setup. For high-resolution experiments involving aberration corrections, it may be advisable to incorporate retinal eye tracking^48–50^ as a means to correct for eye motions^51^. In addition to eye movements, accommodation of the crystalline lens presents another challenge as it can alter the focus shift, thereby introducing errors into the results^52,53^. Furthermore, pupillary reflexes can induce vignetting of the stimulus light. To mitigate these potential issues, both cycloplegia and mydriasis should be maintained throughout the duration of the experiment. Even with usage of Tropicamide residual accommodation is still possible, thus the design of the gaze fixation system should also aim to minimize crystalline lens accommodation and eye movements, particularly those induced by fatigue.

Our psychophysics experiment was designed with an emphasis on robustness, serving as a proof of concept and providing insight into factors that can influence the results. Consequently, we did not consider some of the more subtle factors that could become significant in high-resolution experiments where aberration corrections are applied. One such factor is the specific type of photoreceptor cells (rods and three types of cones) involved in 2P vision in our experiments. Different photoreceptor cell types exhibit varying spectral sensitivities^54^, varying depth locations of their outer segments in the retina^55^, and varying spatial distributions depending on their location within the eye fundus^56^. Given the current design of our experiment, we lack the capability to deliver the stimulus to specific groups of photoreceptor cells or analyze their distinct responses. However, we can speculate based on the spectral bandwidth of our stimulus, which matches the absorption bandwidth of approximately 514–546 nm. We hypothesize that primarily M-cones and rods could be involved in 2P vision in our psychophysics experiment. Additionally, the experimental results obtained from the fovea may suggest a primary involvement of rods. The span of the line stimulus was $5^\circ$ ($\sim$1.5 mm), which covers a length at which the distribution of cones and rods changes with distance from the fovea. All subjects reported a central “gap” in the perceived line of light, wherein the 2P vision intensity gradually diminished until there was no light perception in the center of their visual field. This effect was not reported in the perifovea. Such an observation is in agreement with the known distribution of rods in the eye fundus. The density of rods is practically zero at the center of the fovea and gradually increases with distance from the fovea^56^. One potential counterargument against this hypothesis is the fact that we did not implement full dark adaptation of the subjects. As a result, the rods could have been largely saturated. Clearly, further studies are necessary to fully understand and quantitatively describe the psychophysics of human 2P vision^37,44,57^.

The OCT imaging part of our experiment provided an objective measure of the location of the focus across the depth of the retina. We tested various metrics to determine beam focus location from OCT images. These included metrics like speckle size^58^, quality index^59^ or correlation level between IS/OS and COST projections with focal shifts. However, this selection is not exhaustive, as many other metrics are known in the literature, such as variance, normalized variance, Brenner gradient, Tenengrad gradient, or entropy^60^. Among the metrics we tested, image contrast emerged as a practical indicator of focus position while maintaining simplicity of numerical computations. The image contrast metric can be effectively employed in *en face* OCT images of retinal layers that contain distinct structural features, such as vessels or the photoreceptor mosaic. In these layers, the contrast vs. focal shift curve exhibits an unambiguous peak corresponding to the layer’s position when the light beam is focused accurately. Conversely, this metric is not well-suited for layers that lack specific visible features (only showing speckle patterns) or for areas with a low signal-to-noise ratio (for instance, areas within the shadows cast by vessels). An example is the *en face* image of IS/OS junction near the center of the fovea (Fig. 4). In the area where only the speckle pattern was visible, the contrast graph displayed a very broad distribution with a maximum that was either absent or poorly defined. On the other hand, where the photoreceptor mosaic was discernible, the image contrast graphs exhibited well-defined peaks.

In the case of the image contrast metric, the precision of focus localization is dictated by 1P axial PSF. Without aberration correction in the imaging system, the PSF can be impacted by subject-specific ocular aberrations, potentially accounting for the observed variability in the shapes and widths of contrast distributions, as demonstrated in Fig. 6. The resolution limits of this method, in an idealized aberrations-free imaging system, can be inferred from the 1P PSF. Equation S3 sets the limit of our high-resolution imaging setup as ${\Delta \text {z}_{\text {HWHM}}^{(\text {1P,G})}=17.1}$ µm for ${\text {d}_{\text {cornea},1/\text {e}^2}^{(f150)}=4.8\,\text {mm}}$ beam spot diameter at the cornea. Equation S14 predicts the resolution limit as ${\Delta \text {z}_{\text {HWHM}}^{(\text {1P,A})}=22.7}$ µm in case of uniform illumination of 7 mm diameter pupil (Table 2).

As a final point, it should be noted that the accuracy of the results reported throughout this paper, as indicated by the provided significant digits, is dictated by standard errors derived from calibrations of the experimental setup. The standard error of the focal shift in the retina, determined by the collimator motor step, is 1.1 µm. The standard error of axial locations in the OCT images, obtained through the calibration of the OCT image pixel size, is 1.5 µm (in tissue).

Our results demonstrate collocation of the peak intensity of 2P vision and the position of the outer photoreceptor segments in OCT images, which provides an in vivo localization of visual photopigments in OCT images of the retina as possible reference for unambiguous identification of other retinal layers or depth-locations. The resolution of the results is primarily limited by the aberration-limited 2P point spread function in the case of 2P vision and by the 1P point spread function in the case of image contrast metric used in OCT images. Our experiments indicate that with improved imaging resolution, the localization of 2P vision in the retinal depth becomes more accurate. This suggests that correcting aberrations in the experimental setup could improve the accuracy of the results and potentially help resolve the controversy regarding the anatomical attributions of bands 2 and 3. For example, if the 2P vision peak were to lie symmetrically between the OCT contrast peaks of bands 2 and 3, it could indicate that these bands correspond to the IS/OS junction and the COST, respectively. However, if the 2P vision peak were offset towards the contrast peak of band 2, it would suggest that band 2 might correspond to the photoreceptor’s inner segment ellipsoid (ISe)^14,24^. Currently, the resolution of our experiment does not allow us to make this claim.

2P vision as a tightly spatially localized phenomenon is one of possible tools to give experimental answers to questions regarding subtle effects like the role of waveguiding properties of the photoreceptor cells in light capturing efficiency by chromophores in their outer segments, the role of photoreceptor cells waveguiding in 2P vision and other optical and visual phenomena occurring in the retina. However, there are also many confounding factors which can skew such studies and render their results unusable. Such factors should be identified prior to more advanced studies to avoid methodological and experimental mistakes. Reduction of aberrations can provide narrower localization of the 2P light stimulus, for example, in psychophysics experiments. However, it also poses a few challenges. The tighter the localization of the stimulus transversely and axially, the more care needs to be taken to eliminate factors that may confound the experiments using 2P vision such as eye motions, accommodation of the crystalline lens or the pupillary reflex to light (stimulus or ambient illumination). These observations suggest the following improvements in experiments using 2P vision: the use of adaptive optics to improve the localization of 2P absorption; the use of eye tracking to maintain the localization of 2P stimulus in the desired transverse and axial position in the retina; a fixation system to minimize the accommodation and eye motion; careful control of stimulus and ambient light in experiments; and stimuli matching the distribution of the photoreceptor cells of interest in the retina.

## Methods

### Human subjects preparation

Four volunteers (two males and two females, ages between 42 and 44) were recruited for the study. Three are emmetropes and one is a low myope (spherical correction of − 1.5 D and cylindrical correction of − 0.6 D). The volunteers have no history of eye diseases. Preparation of the subjects included cycloplegia and mydriasis induced by topical application of 1% solution of Tropicamide, repeated every 30 min, to avoid light vignetting by the eye pupil and to minimize crystalline lens accommodation. Photographs of the subjects’ irises were taken with the pupil viewing camera of our experimental setup, from which pupil diameters were measured to be 7 mm in all subjects. The light power at the cornea was 1.8 mW in OCT imaging and in psychophysics experiments, which is in compliance with the recommendations for permissible laser light exposure limits for the human eye^46,61^. The safety limits were calculated for repeated B-scan imaging (one-line scanning) which can be performed continuously over the same location in the retina for 25 min. The subjects were asked to blink between the OCT data acquisitions and psychophysics tests, and allowed to move their gaze. Therefore, the continuous one-line scanning was effectively performed for no longer than a few minutes at a time. Longer breaks (5–10 min) were taken every 30 min. for re-application of Tropicamide.

Two separate experimental sessions were held on two separate days for each subject to test and image either the parafoveal (including the fovea) or perifoveal ($6.5^\circ$ nasal and $3^\circ$ superior from the fovea) region of the retina. The psychophysics experiments were performed first, followed by OCT imaging in the same experimental session. The duration of the psychophysics tests ranged from $\sim$1 to 3 h depending on the subject. The duration of OCT imaging less than 1.5 h. During the experiments the lights in the room were turned off, however the subjects were not fully dark adapted. The study adhered to the tenets of the Declaration of Helsinki and was approved by the Bioethics Committee of Nicolaus Copernicus University in Torun, Poland. Informed consent was obtained from all participants.

**Table 3:** Parameters of the swept-source OCT system used for retinal imaging.

Laser sweep frequency	1.6 MHz	
Center wavelength	1060 nm	
Spectral bandwidth	65 nm	
Axial imaging resolution in tissue	6 µm	
Light power at the cornea	1.8 mW	
Optical fiber mode field diameter	6.2 µm	
	L_3_	L’_3_
Focal length	150 mm	75 mm
Beam spot diameter at the cornea	4.8 mm	2.4 mm
Beam spot diameter at the retina	4.8 µm	9.6 µm
Depth of focus	34 µm	136 µm

Transverse and axial beam focus parameters are calculated for Gaussian beam propagation in aberration-free eye.

### OCT imaging

**Figure 7:**
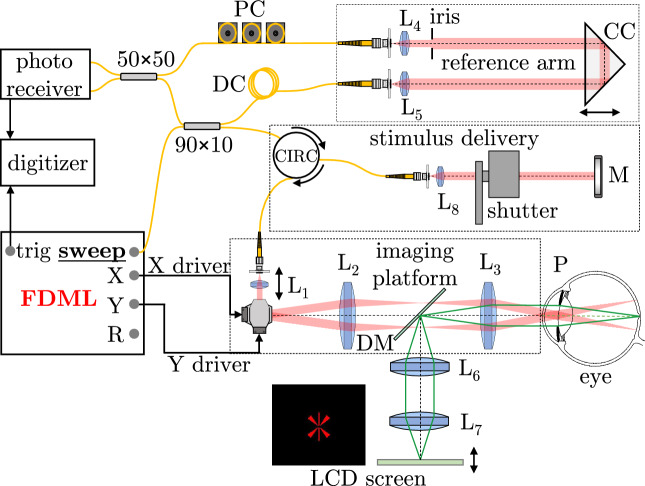
Layout of the FDML SS-OCT system. *PC* polarization controller, *DC* dispersion compensation, *CC* corner cube, *CIRC* optical circulator, *M* mirror, *DM* dichroic mirror, *L* lenses, *P* pupil plane.

Retinal imaging was performed with a custom-made Fourier-domain swept-source OCT system incorporating a Fourier-domain mode-locked (FDML) laser operating at 1.6 MHz A-scan rate. The system schematic is shown in Fig. 7. Our swept-source OCT system used a Mach-Zehnder interferometer layout in which we incorporated a detachable light stimulus delivery module which is used only in psychophysics experiments (Human psychophysics). The collimator of the sample arm (L_1_) is mounted in a stepper motor translation stage to facilitate automated beam focus shift in the retina. The LCD screen is used to present eye fixation mark and is also used in psychophysics experiments (Psychophysics task).

The system parameters are given in Table 3. Assuming an idealized case of an untruncated Gaussian beam propagating in the eye with 60 dioptre optical power and no aberrations, the 1/e^2^ beam spot diameter in the retina would be 4.8 µm, Rayleigh range (half width at half maximum of the Lorentzian function describing axial light distribution in Gaussian beams) would be 17.1 µm resulting in the depth-of-focus DoF$\approx$34 µm. However, uncorrected wavefront aberrations introduced by the eye deteriorate the imaging resolution of OCT imaging. Given the visibility of individual cone photoreceptor cells in our OCT images of the perifovea, we can assume that the beam spot radius at the retina is not larger than $\sim$10 µm. The axial OCT imaging resolution depends on the spectral bandwidth of the light contributing to the interference signal and it is $\sim$6 µm (in tissue).

Three-dimensional OCT data were acquired for a set of 31 different focus positions in the retina (described in details in Psychophysics task section). At least 6 OCT data sets were acquired at each focus position (more if significant eye motion occurred during the imaging). No co-registration between volumes and no volume averaging was performed. The parameters of the OCT scan protocol are given in Table 4.

**Table 4:** Retinal scanning parameters.

Points per A-scans	2304
A-scans	800
B-scans	800
Field of view	$5^\circ \times 5^\circ$ (~1.5 mm $\times$ 1.5 mm)
Imaging time	1 s

### Human psychophysics

Human psychophysics experiments were performed using the same imaging system that was used for retinal OCT imaging (Fig. 7). In this case it is used to deliver the stimulus to the retina. The galvanometer scanners are used to perform a horizontal scan. The subjects perceived the stimulus as a horizontal line with a span of $5^\circ$ ($\sim$1.5 mm). The imaging system was augmented with a stimulus delivery optical setup connected to the imaging platform via a fiberoptics circulator. The role of this extension was to provide a blinking stimulus^62^ by means of a beam shutter. The stimulus has 1 Hz frequency with light turned on for 0.2 s and off for 0.8 s. A flicker is implemented to maintain subjects attention and minimize fatigue from the prolonged stimulus.

The light from the FDML laser serves as a near infrared stimulus for 2P triggered vision. During the 0.625 µs sweep duration, corresponding to 1.6 MHz sweep frequency, the retina is exposed continuously to subsequent wavelengths of light (65 nm bandwidth centered at 1060 nm). Therefore, our light source can be effectively treated as a CW laser. According to Denk et al.^63^, the 2P absorption rate is defined as the number of photons $n_a$ absorbed in the 2P absorption process per chromophore per unit time as $\text {TPAR} \approx \frac{P_0^2 \delta }{\tau _p f_p} \left( \frac{NA^2}{2\hbar c \lambda }\right) ^2$, where $P_0$ [J/s] is the power of light in the beam, $\delta$ [s$\cdot$m$^4$] is the 2P absorption cross-section, $\tau _p$ [s] is the pulse duration, $f_p = 1/T_p$ [1/s] is the pulse repetition frequency, $T_p$ is the pulse repetition period, $NA$ is the numerical aperture of the lens, $\hbar$ [J$\cdot$s] is the reduced Planck constant, $c$ [m/s] is the speed of light, and $\lambda$ [m] is the wavelength. Setting $f_p = 1/\tau _p$ at the limit of continuous wave laser operation (pulse repetition period equal to the pulse duration), i.e., duty cycle = 1, one can derive $\text {TPAR} \approx P_0^2 \delta \left( \frac{NA^2}{2\hbar c \lambda }\right) ^2$. In our setup, the measured power of light entering the eye is 1.8 mW at 1060 nm wavelength, which is an average over the light spectrum emitted by the light source (65 nm bandwidth). However, the amount of light reaching the chromophores depends on the optical parameters of the ocular media (loss of light e.g., due to scattering and absorption), and is subject dependent. The eye numerical aperture (given by the beam spot diameter at the cornea) is in our case: $NA = 0.14$. The estimated 2P absorption cross-section of rhodopsin at 950–1050 nm (ex vivo, according to Gholami et al.^64^) is $\delta = 260 \times 10^{-50}$ cm$^4$ s per photon per molecule. Given these values, in a rough approximation, the number of photons absorbed per chromophore per unit time in 2P absorption is $\text {TPAR} \approx 0.007 \, \text {[1/s]}$.

### Psychophysics task

The stimulus protocol consisted of presenting the subjects with a horizontal line of near infrared light (center wavelength 1060 nm) with different amount of defocus in subsequent trials. Defocus was implemented by shifting the collimator (L_1_ in Fig. 7) to 31 positions in 21 µm steps. Each focus position was repeated 6 times giving a total of 186 trials presented in a random order. The corresponding focus positions range in the retina was ($-283$; 283) µm relative to the initial focus position (Table 5). The initial focus position was set by the subjects prior to psychophysics experiments and OCT imaging. Each subject adjusted the position of the collimator so that the perceived stimulus light intensity was the strongest. That position was set as the origin ($\Delta {}z=0$) from which the collimator shifts were implemented. The setting of the origin was the same for OCT imaging and for the psychophysics experiments.

The stimulus was visible to the subjects against the LCD screen in the eye fixation path. The task was to match the intensity of the green background of the screen (hue-saturation-intensity color representation) to the perceived intensity of the stimulus (a contrast matching task) in 8-bit scale (0–255) via a remote keypad. In each of the 186 trials, the focus was moved and the LCD screen intensity was set to 0 (black). The subjects were instructed to focus their gaze at a red fixation mark (a Maltese cross) and take as much time as necessary to match the screen intensity to the perceived stimulus light intensity. The line was dim even at the best focus and the intensity values were usually below 50. Data presented in the paper was normalized to 1. In our experiments absolute values of the perceived light intensity are not important. Of interest are the focus shifts at which the subjects experience the strongest 2P vision. They indicate the focus position in the retina at which visual photopigments are located allowing for the most efficient 2P chromophore isomerization^30^.

**Table 5:** Parameters of the psychophysics experiments.

Stimulus light wavelength	1060 nm	1060 nm	520 nm
Lens L_3_ focal length	150 mm	75 mm	150 mm
Collimator step size	21 µm	49 µm	21 µm
Number of collimator steps	31	31	39
Focus shift range in the retina	±283 µm	±613 µm	±377 µm

### Impact of optical resolution and wavelength on light perception

In one of the volunteers (Subject 2) we have performed additional psychophysics experiments to demonstrate how the light perception differs depending on optical resolution in 2P vision as well as between 2P and 1P vision.

To demonstrate the effect of optical resolution on stimulus delivery and in consequence on the psychophysics experiments results, we replaced lens L_3_, f_L3_=150 mm with a lens of focal length f_L3’_=75 mm in our imaging setup. This changes the focusing of light at the retina (Table 3) and changes the 2P-triggered light perception (Fig. 6). Each of the 31 collimator shifts was set to 49 µm to cover a wider range of focus shifts in the retina ($-613$, 613) µm, to capture the “tails” of the 2P light perception plots.

To compare the light perception in two-photon and one-photon triggered vision we have replaced the near infrared (1060 nm) with green (520 nm) light stimulus. The dichroic mirror was replaced with a beamsplitter to allow the visible light to pass through towards the eye. The focal length of lens L_3_ was f_L3_=150 mm. The optical-fibers guiding 1060 nm wavelength light in a single (fundamental) mode are multimode fibers for visible light. However, the stimulus is still perceived in our setup as a single line of light without any structure of the illumination that would suggest dominance of any specific higher order mode propagating in the fiber. The collimator shift steps were 21 µm, the number of steps was 39 to cover a range of ($-377$, 395) µm focus shifts in the retina.

In 1P vision experiment the initial collimator position set by the subject for the maximum light perception intensity was different than in the 2P vision experiment. This is due to chromatic shift of focus in the eye. But also changes in light intensity were smaller with defocus making it difficult to set the collimator shift to maximum light intensity. For the purpose of presentation we shifted the 1P vision and 2P vision plots so that zero focus shift matches their respective maxima (Fig. 1). This operation is justified since in this case we compare the width of the plots rather than the peak positions.

In both cases the psychophysics experiment was performed in a similar way as described in previous sections. The collimator shifts and focus shift ranges in the retina are summarized in Table 5.

### Image and data processing

OCT *en face* projections were generated by axial averaging of five adjacent C-scans (depth slices) selected from 3D data sets, centered at a depth at which the anatomical layer of interest was located (location of the signal intensity peak of that layer). Prior to *en face* image generation B-scans were corrected for the curvature of retinal pigment epithelium (“flattened” to the RPE) as described in Ref.^65^.

**Figure 8:**
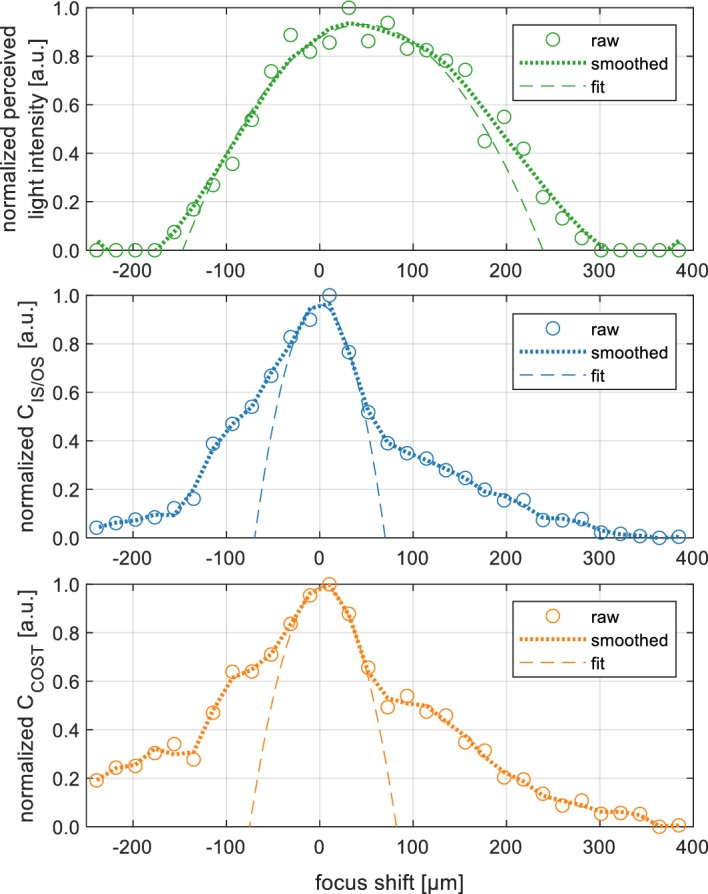
Example data from Subject 3 smoothed with Savitzky-Golay filter and subsequently fitted with a parabola. Green - normalized perceived light intensity (green). Blue—IS/OS junction contrast. Orange—COST layer contrast.

Three retinal layers were chosen for the analysis. Inner/outer photoreceptor junction (IS/OS) was localized automatically by detecting the second outer retinal band peak in a volume-averaged A-scan (mean axial signal intensity distribution graph). A distance from IS/OS junction to cone outer segments tips layer (COST) and to the junction between Inner Nuclear Layer (INL) and Outer Plexiform Layer (OPL) were found manually in a single data set for each subject. *En face* images of COST layer and INL/OPL junction were then generated from all acquired 3D data sets at these pre-determined constant depth offsets from IS/OS junction. COST and IS/OS junction were selected for the analysis since the expected location of visual photopigments falls between these layers. In addition, INL/OPL junction was selected to test if the used image quality metric correctly shows the location of focus in the retina, as expected from known focus shifts controlled by the stepper motor with a collimator attached to it. The INL/OPL junction contains the vasculature of the deep capillary plexus, which is a structure well visible in *en face* projections and suitable for image quality analysis.

To determine the depth position of the focus in the retina from the OCT data, we used an image contrast metric calculated in *en face* projections as $C = \frac{\upsigma }{\langle I \rangle }$, where $\upsigma$ is the standard deviation and $\langle I \rangle$ is the mean intensity of the image pixels within regions of interest (ROIs, Figs. 2 and 4). When in focus, image features like cones mosaic or capillary vessels have high contrast. When focus moves away from a specific layer visualized in *en face* projection, the imaging contrast deteriorates.

To account for the eye motion occurring between the acquisition of 3D data sets, ROIs were selected so that the analyzed areas were within all collected data sets (bold outline in Fig. 2 and two solid-line rectangles in Fig. 4). The data sets were corrected for motion using a reference frame and a cross-correlation algorithm^66^ to always calculate contrast in regions containing the same retinal features. The selection of ROIs in the parafovea were dictated by the anatomy of the retina. The foveal zone outlined with a red circle in Fig. 4) does not contain features suitable for image contrast analysis (neither vessels nor photoreceptor mosaics are visible in the acquired data sets) and was excluded from the analysis. We additionally divided the ROIs into smaller regions (12 in the perifoveal and 6 in the parafoveal images) and calculated the contrast individually in each subregion to check for location-specific differences. Contrast graphs of the IS/OS junction and COST layer images (2nd and 3rd outer retinal bands) differ in their particular shapes but the peak positions (maximum contrast) coincide (are well within the DoF) for all subregions in the perifovea and in the parafovea. Therefore, for simplicity of presentation, we demonstrate the results of contrast calculations in the entire ROI for each subject.

Contrasts were also calculated for the INL/OPL junction in the perifovea as a verification of the used experimental and data analysis methods. *En face* images of INL/OPL junction visualize capillary vessels providing suitable features to test the image contrast metric behaviour with defocus and to check distances between retinal layers measured from contrast graphs against their distances measured directly from OCT images.

To show the contrast graphs alongside 2P vision intensity graphs we normalized both plots to 1. To find the position of the maximum, the data was smoothed using Savitzky-Golay filter. The window size was empirically adjusted for each case individually to account for local minima and to account for the individual peak widths. Specifically, the image contrast graphs had a narrower peak when the photoreceptor mosaics were visible at optimal focusing and wider peaks when the mosaic was no longer visible due to a large defocus. After smoothing of the data, parabolic function fit was performed. The position of the maximum of the parabolic function was used as the position of the maximum contrast. Figure 8 shows examples of the smoothing and parabolic fits to the data sets.

### Supplementary Information

Supplementary Information.

## Data Availability

The volume of raw optical coherence tomography data generated from our experiments exceeds the capacity limitations of standard online repositories. However, data from our two-photon vision experiments, as well as the processed contrast curves, are available from the authors upon a justified request.
