# Supplementary material for: In vivo identification of the retinal layer containing photopigments in OCT images through correlation with two-photon psychophysics
Source: Sci Rep. 2024 Jul 4;14:15459. doi: 10.1038/s41598-024-65234-7 (PMC11224378; doi:10.1038/s41598-024-65234-7)
Supplement: Supplementary file 1 — Supplementary Information. [file 41598_2024_65234_MOESM1_ESM.pdf]

Supplementary discussion

The results provided in Table 2 of the Discussions were calculated using 1P and 2P point spread functions. For clarity, here we provide the formulas used for the computations.

Should aberrations be corrected in our experiment, i.e. diffracition limitted imaging should apply, Gaussian beam imaging would be achieved. This is due to the light delivery via single-mode optical fiber and the fact that the light beams are not significantly truncated by either the optical elements of the experimental setup or the eye pupil (eye pupil diameters were *circa* 7 mm in all subjects). The amplitude PSF of a Gaussian beam is given by:

$$\mathrm{U_G(r,z) = U_0 \frac{w_0}{w(z)} \exp\left[-\frac{r^2}{w(z)^2}\right] \exp\left\{-i\left[kz + \frac{kr^2}{2R(z)} - \psi(z)\right]\right\}}, \tag{S1}$$

where $\mathrm{U_0}$ is the input light amplitude, R is the radius of the wavefront curvature, ψ is the Gouy phase, r is the radial coordinate measured from the optical axis at the focal plane, z is the axial coordinate measured from the focal plane, i is the imaginary unit, $\mathrm{w(z)}$ is the radius of the beam spot at distance z from the waist (focus), $\mathrm{w_0}$ is the radius of the beam waist measured at $\mathrm{1/e^2} \approx 0.135$ peak light intensity, k is the wavenumber where $\mathrm{k} = 2\pi/\lambda$, λ is the wavelength in medium with $\lambda = \lambda_v/n$, λ_v is the wavelength in vacuum, and n is the index of refraction for λ_v. The intensity PSF in the focal plane ($\Delta \mathrm{z} = 0$) follows, under non-coherent illumination, a well-known Gaussian distribution:

$$\mathrm{PSF_{G,T}^{(1P)} = |U_G(r,0)|^2 \sim \exp\left[-\frac{2r^2}{w_0^2}\right]}. \tag{S2}$$

In our case, we are primarily interested in the axial PSF, i.e., along the optical axis ($\mathrm{r} = 0$). It is given by the Lorentz function, also known as the Cauchy distribution:

$$\mathrm{PSF_{G,A}^{(1P)} = |U_G(0,z)|^2 \sim \left|\frac{w_0}{w(z)}\right|^2 = \frac{1}{1+(z/z_R)^2}}, \tag{S3}$$

where $\mathrm{z_R}$ denotes the Rayleigh range, and $\mathrm{2z_R = DoF}$ (depth of focus), subscripts G,T and G,A indicate transverse and axial Gaussian PSFs. Equation S2 defines the measure of the transverse beam spot size as the Gaussian beam waist radius at $\mathrm{1/e^2} \approx 0.135$ peak intensity of the PSF. For a given focal length of the focusing lens (in our case, it corresponds to the focal length of the eye $\mathrm{f_{eye}} \approx 1/60$ dioptres $\approx$ 17 mm), and collimated beam waist $\mathrm{w_{col}}$, this can be calculated as:

$$\mathrm{r_{G,1/e^2}^{(1P)} = w_0 = \frac{\lambda}{\pi} \frac{f_{eye}}{w_{col}}}, \tag{S4}$$

where $\mathrm{w_{col}}$ represents the radius at the waist of the collimated beam. The transverse spot radius can be also calculated at 0.5 light intensity drop from the peak value of the normalized PSF (half-width at half-maximum, HWHM) as:

$$\mathrm{r_{G,HWHM}^{(1P)} = \frac{1}{2}\sqrt{2\ln 2}\, w_0 \approx 0.59\, w_0}. \tag{S5}$$

Equation S3 defines the Rayleigh range (HWHM of the Lorentz function)

$$\mathrm{\Delta z_{G,HWHM}^{(1P)} = z_R = \frac{\pi}{\lambda}\, w_0^2} \tag{S6}$$

and can be used to calculate the axial half-span at $\mathrm{1/e^2}$ light intensity of the peak value:

$$\mathrm{\Delta z_{G,1/e^2}^{(1P)} = \sqrt{e^2-1}\, z_R \approx 2.53\, z_R}. \tag{S7}$$

For Gaussian beams, the transverse and axial 2P intensity PSFs can be calculated as squares of Eq.S2 and S3. The spot radii at 0.5 and $\mathrm{1/e^2}$ peak intensity are given as:

$$\mathrm{r_{G,HWHM}^{(2P)} = \frac{1}{2}\sqrt{\ln 2}\, w_0 \approx 0.42\, w_0}, \tag{S8}$$

$$\mathrm{r_{G,1/e^2}^{(2P)} = \frac{1}{\sqrt{2}}\, w_0 \approx 0.71\, w_0}, \tag{S9}$$

$$\Delta z^{(2P)}_{G,HWHM} = \sqrt{(\sqrt{2}-1)}\ z_R \approx 0.64\ z_R, \tag{S10}$$

$$\Delta z^{(2P)}_{G,1/e^2} = \sqrt{(e-1)}\ z_R \approx 1.31\ z_R. \tag{S11}$$

When AO is used, the collimated beam of light typically overfills the pupil of the eye to achieve approximately uniform light distribution. If we assume an idealized case of uniform illumination of the eye pupil (rectangular, or "top-hat" function), then the amplitude PSF describing light distribution near the focus is given by three-dimensional Airy function[43]:

$$U_A(r,z) = -i\frac{ka^2}{f_{eye}}U_0\exp(ikz)\int_0^1 J_0\left(\frac{ka}{f_{eye}}r\rho\right)\exp\left(-i\frac{ka^2}{2f^2_{eye}}z\rho^2\right)\rho d\rho, \tag{S12}$$

where J_0 denotes Bessel function of the first kind and 0th order, a represents the aperture (eye pupil) radius, ρ is the normalized, radial coordinate at the aperture, $N.A. = a/f_{eye}$ denotes the numerical aperture the lens (of the eye).

Equation S12 can be reduced to transverse and axial intensity PSFs (non-coherent illumination case):

$$PSF^{(2P)}_{A,T} = |U_A(r,0)|^2 \sim \left|\int_0^1 J_0\left(\frac{ka}{f_{eye}}r\rho\right)\rho d\rho\right|^2 = \left|\int_0^1 J_0\left(\frac{2\pi}{\lambda}r\rho N.A.\right)\rho d\rho\right|^2, \tag{S13}$$

$$PSF^{(2P)}_{A,A} = |U_A(0,z)|^2 \sim \left|\int_0^1 \exp\left(-i\frac{ka^2}{2f^2_{eye}}z\rho^2\right)\rho d\rho\right|^2 = \left|\int_0^1 \exp\left(-i\frac{\pi}{\lambda}z\rho^2 N.A.^2\right)\rho d\rho\right|^2, \tag{S14}$$

where subscripts A,T and A,A indicate transverse and axial Airy PSFs. Typically the first minima of these functions (eqs. S13 and S14) are used to define the radius and axial span of the focused beam spot size:

$$PSF^{(1P)}_{A,T}(1.22\pi,0) = 0 \rightarrow r_{A,1^{st}min.} = 1.22\frac{\lambda}{2N.A.}, \tag{S15}$$

$$PSF^{(1P)}_{A,A}(0,2\pi) = 0 \rightarrow \Delta z_{A,1^{st}min.} = \frac{2\lambda}{N.A.^2}, \tag{S16}$$

but other light intensity levels can be used to define them as well. For example, tabulated functions or numerical computations can be used to find the radius and axial span of the focused beam spot at 0.5 or $1/e^2$ of the peak intensity of the normalized PSFs:

$$PSF^{(1P)}_{A,T}(0.51\pi,0) = 0.5 \rightarrow r^{(1P)}_{A,HWHM} \approx 0.26\frac{\lambda}{N.A.}, \tag{S17}$$

$$PSF^{(1P)}_{A,T}(0.82\pi,0) = 1/e^2 \rightarrow r^{(1P)}_{A,1/e^2} \approx 0.41\frac{\lambda}{N.A.}, \tag{S18}$$

$$PSF^{(1P)}_{A,A}(0,0.89\pi) = 0.5 \rightarrow \Delta z^{(1P)}_{A,HWHM} \approx 0.89\frac{\lambda}{N.A.^2}, \tag{S19}$$

$$PSF^{(1P)}_{A,A}(0,1.4\pi) = 1/e^2 \rightarrow \Delta z^{(1P)}_{A,1/e^2} \approx 1.4\frac{\lambda}{N.A.^2}. \tag{S20}$$

In the case of 2P light absorption, the transverse and axial 2P PSFs are squares of Equations S13 and S14 [34]. Again by resorting to tabulated functions or numerical computations, we can find the radii and axial spans at $1/e^2$ and 0.5 of the peak light intensity of the focused beam:

$$\mathrm{PSF}^{(2P)}_{A,T}(0.37\pi,0) = 0.5 \rightarrow \mathrm{r}^{(2P)}_{A,HWHM} \approx 0.18\,\frac{\lambda}{\mathrm{N.A.}}, \tag{S21}$$

$$\mathrm{PSF}^{(2P)}_{A,T}(0.61\pi,0) = 1/\mathrm{e}^2 \rightarrow \mathrm{r}^{(2P)}_{A,1/e^2} \approx 0.305\,\frac{\lambda}{\mathrm{N.A.}}, \tag{S22}$$

$$\mathrm{PSF}^{(2P)}_{A,A}(0,0.64\pi) = 0.5 \rightarrow \Delta \mathrm{z}^{(2P)}_{A,HWHM} \approx 0.64\,\frac{\lambda}{\mathrm{N.A.}^2}, \tag{S23}$$

$$\mathrm{PSF}^{(2P)}_{A,A}(0,1.05\pi) = 1/\mathrm{e}^2 \rightarrow \Delta \mathrm{z}^{(2P)}_{A,1/e^2} \approx 1.05\,\frac{\lambda}{\mathrm{N.A.}^2}. \tag{S24}$$

The assumptions made so far regarding the 2P absorption PSFs have been based on an infinitely thin absorbing retinal layer. However, in reality, light-absorbing chromophores are distributed throughout the outer segments of the photoreceptor cells, which are approximately 30 μm in length [11]. Therefore, a more accurate representation of our experiment would involve convolving the axial 2P absorption PSF with a function that encapsulates the axial distribution of these chromophores. If we operate under the premise that the probability of 2P absorption is constant regardless of the location within the photoreceptor outer segments, then this distribution can be suitably represented by a rectangular function.

The results of calculations of the focused beam spot sizes with parameters characterizing our experimental setup are given in Table 2.
